# Exosomes derived from atorvastatin-pretreated MSC accelerate diabetic wound repair by enhancing angiogenesis via AKT/eNOS pathway

**DOI:** 10.1186/s13287-020-01824-2

**Published:** 2020-08-12

**Authors:** Muyu Yu, Wei Liu, Junxian Li, Junxi Lu, Huijuan Lu, Weiping Jia, Fang Liu

**Affiliations:** 1 Department of Endocrinology and Metabolism, Shanghai Diabetes Institute, Shanghai Key Laboratory of Diabetes Mellitus, Shanghai Clinical Center for Diabetes, Shanghai Jiao Tong University Affiliated Sixth People’s Hospital, Shanghai, 200233 China; 2 Spine Center, Department of Orthopaedics, Shanghai Changzheng Hospital, Second Military Medical University, Shanghai, 200003 China

**Keywords:** Exosome, Mesenchymal stem cell, Atorvastatin, Diabetic wound, Angiogenesis

## Abstract

**Background:**

Mesenchymal stem cell (MSC)-derived exosomes emerge as promising candidates for treating delayed wound healing in diabetes due to the promotion of angiogenesis. Preconditioned MSC with chemical or biological factors could possibly enhance the biological activities of MSC-derived exosomes. The purpose of this research focused on whether exosomes derived from the bone marrow MSC (BMSC) pretreated with atorvastatin (ATV), could exhibit better pro-angiogenic ability in diabetic wound healing or not and its underlying molecular mechanism.

**Methods:**

We isolated exosomes from non-pretreated BMSC (Exos) and ATV pretreated BMSC (ATV-Exos) and evaluated their characterization by transmission electron microscopy (TEM), nanoparticle tracking analysis (NTA) and Western blotting. In vivo, we made full-thickness skin defects in streptozotocin (STZ)-induced diabetic rats and the defects received multiple-point injection with PBS, Exos, or ATV-Exos. Two weeks later, histological analysis was conducted to evaluate the impact of different treatments on wound healing and the neovascularization was measured by micro-CT. In vitro, cell proliferation, migration, tube formation, and vascular endothelial growth factor (VEGF) secretion were measured in human umbilical vein endothelial cells (HUVEC). The role of miRNAs and AKT/eNOS signaling pathway in the promoted angiogenesis of ATV-Exos were assessed with their inhibitors.

**Results:**

No significant difference in morphology, structure, and concentration was observed between ATV-Exos and Exos. In STZ-induced diabetic rats, ATV-Exos exhibited excellent abilities in facilitating the wound regeneration by promoting the formation of blood vessels compared with Exos without influencing liver and kidney function. Meanwhile, ATV-Exos promoted the proliferation, migration, tube formation, and VEGF level of endothelial cells in vitro. And AKT/eNOS pathway was activated by ATV-Exos and the pro-angiogenic effects of ATV-Exo were attenuated after the pathway being blocked. MiR-221-3p was upregulated by ATV-Exos stimulation, and miR-221-3p inhibitor suppressed the pro-angiogenesis effect of ATV-Exos.

**Conclusions:**

Exosomes originated from ATV-pretreated MSCs might serve as a potential strategy for the treatment of diabetic skin defects through enhancing the biological function of endothelial cells via AKT/eNOS pathway by upregulating the miR-221-3p.

## Background

Diabetes is a multifaceted metabolic disease, and up to 20% diabetes have developed diabetic wound all over the world [1]. Despite a lot of conventional therapies for chronic wounds that have been applied, the recovery rates remain low and approximately 28% of these patients have to undergo lower extremity amputation [2]. Among diverse factors influencing wound repair, angiogenesis occupies a critical position, which results in the delivery of nutrition and oxygen to the wound sites and in turn augments fibroblasts proliferation, collagen synthesis, and re-epithelialization [3, 4] while the impaired angiogenesis of diabetic patients plays a critical role in their delayed wound healing. Hence, the novel therapies to stimulate angiogenesis are highly promising for treating a diabetic wound. Mesenchymal stem cells (MSCs) are multipotent, nonhematopoietic adult stem cells, being able to significantly increase the regenerative capacity of many tissues [5]. Accumulating evidences have confirmed the crucial role of MSC in the vasculogenesis of many pathological processes, such as wound healing, bone repair, and myocardial infarction by promoting local revascularization via secreting angiogenic growth factors [6–8].

Recently exosomes have been proved to act as a class of the paracrine factor to mediate the function between MSC and target cells in wound healing and tissue repair [9, 10]. Exosomes, small extracellular vesicles in a diameter ranging from 30 to 200 nm, are produced by many types of cenlls via the inward budding of late endosomes and fuse with the plasma membrane. In the past few decades, exosomes have been regarded as a vital cell-cell communication tool, delivering cargoes (miRNA, mRNA or proteins) from parental cells to recipient cells, which influence intracellular signaling and various biological processes [11, 12]. MSCs secrete plentiful of exosomes, and many regenerative abilities have been proved to be ascribed to the MSC-secreted exosomes, instead of stem cells themselves in the conventional view [13]. For instance, the exosomes derived from MSCs were reported to induce angiogenesis of human umbilical vein endothelial cell (HUVEC) in a dose-dependent manner in vitro [14]. Additionally, enhanced generation of developed neovessels and their expedited collagen maturation in wound sites were observed when cells treated with pluripotent stem cell secreated exosomes [15]. Compared with MSCs, their exosomes present obvious advantages such as excellent stability, limited immune rejection, convenient administration, and easy internalization into recipient cells [16]. On the basis of these findings as well as the efficient and inexpensive exosome extraction with the development of isolation methods in the last few years, especially the ultracentrifugation [17], MSC-Exos, the cell-free particles, may act as a promising candidate as a pro-angiogenic therapy of wound healing.

Previous studies have suggested that precondition MSC with physical, chemical, and biological factors are effective approaches to enhance the biological activities of MSC-Exos, raising their repairing efficacy in tissue engineering and regeneration medicine. For example, exosomes extracted from IL-1β-pretreated MSC stimulated more IL-10 and TGF-β than exosomes from untreated MSCs [18]. Another study indicated that exosomes derived from dimethyloxalylglycine-preconditioned MSC obviously enhanced bone regeneration via enhanced angiogenesis [19]. Atorvastatin (ATV), a HMG-CoA reductase inhibitor used to reduce the blood lipid in clinical, has been reported to promote tissue regeneration of acute lesions in rats and regulate cell growth-related proteins and cytokines [20]. More importantly, in a rat model of acute myocardial infarction, compared to non-pretreated MSC-exacted exosomes, exosomes derived from ATV-treated MSC (ATV-Exos) were able to enhance the survival, migration, and tube-like structure formation of endothelial cells and the vascular endothelial growth factor (VEGF) level was also increased [21]. These properties of ATV-Exos were conductive to wound healing which has never been verified until now.

Therefore, this study aimed to explore whether the exosomes extracted from MSCs pretreated with ATV could enhance the biological properties of endothelial cells to facilitate angiogenesis in diabetic cutaneous wound healing. Moreover, we further investigated the role of miRNAs and AKT/eNOS pathway involved in the promotion of wound healing of ATV-Exos.

## Methods

### BMSC culture and pretreatment

Human bone marrow MSCs (BMSCs, P4) were obtained from the Chinese Academy of Sciences (Shanghai, China). BMSCs were incubated in α-MEM (Gibco) supplemented with 10% fetal bovine serum (FBS, Gibco) and 1% penicillin-streptomycin (Gibco). For concentration screening, BMSCs were treated in complete culture medium with exosome-free FBS, supplemented with different concentrations of ATV (Selleck, 0.25 μM, 0.5 μM, 1 μM, and 2 μM) for 48 h and subsequently 1 μM was determined as the optimum concentration by cell counting kit-8 (CCK8) method (5% CO_2_ at 37 °C).

### Exosome isolation and identification

Ultracentrifugation was applied in this study to isolate and extract exosomes. When the confluence of BMSC reached about 80%, the supernatant was removed and replenished by serum-free culture medium. The equivalent volume of PBS and ATV was then added into the dishes of BMSC and incubated for 48 h. Subsequently, conditioned medium treated by PBS or ATV was collected before centrifugation at 300 g for 5 min and 2000 g for 20 min. The conditioned medium was then filtered via a filter (Micropore, 0.22 μm) before centrifuged at 100,000 g for 1.5 h twice. Subsequently, the obtained pellets were resuspended with PBS and stored at − 80 °C for further experiments.

For the validation of exosome, the morphology of exosomes was observed by transmission electron microscopy (TEM, JEM-1400, JEOL, Japan). Nanoparticle tracking analysis (NTA) was used for determining the size distribution and particle concentration of exosomes. The specific surface markers of exosome (TSG101, Alix, CD81) were detected via Western blotting.

### Internalization of exosomes

Exosomes derived from PBS- and ATV-treated BMSC were labeled with red fluorescence dye PKH26 (Sigma-Aldrich, Germany). Then, HUVEC was cultured with PKH26-labeled exosomes for 24 h followed by 4% paraformaldehyde fixation for 15 min. After washed by PBS for three times, HUVECs were subjected to phalloidin (5 g/ml) for 1 h for staining the cytoskeleton. Then, after washed by PBS for three times, the nuclei were stained by 2 mg/mL DAPI at room temperature for 5 min. Finally, HUVECs were observed via a laser confocal microscope (Leica, Solms, Germany).

### HUVEC culture and treatment

HUVECs were acquired from the Chinese Academy of Sciences (Shanghai, China). Endothelial cell medium (ECM, Sciencell) was applied to culture HUVECs supplemented with 5% FBS (Sciencell) and 1% endothelial cell growth supplement (ECGS) (Gibco). Then, HUVECs were incubated in low glucose (LG, 5.56 mM glucose+ 27.44 mM mannitol), high glucose (HG, 33 mM glucose), HG + Exos (50 μg/mL), or HG + ATV-Exos (50 μg/mL) medium (5% CO_2_ at 37 °C). HUVECs were pretreated with AKT inhibitor LY294002 (10 μM) to explore the role of AKT/eNOS pathway involved in ATV-Exos mediated angiogenesis.

### Cell viability assay

The cell viability and proliferation were evaluated by CCK8 test. HUVEC was seeded onto 96-well plate at a density of 2 × 10^3^ cells/well and treated with LG, HG, HG + Exos (50 μg/mL), and HG + ATV-Exos (50 μg/mL). CCK-8 solution (10 μl per well) was added into the culture medium of each well, and after that, the 96-well plate was put into the cell incubator at 37 °C for 2 h. Finally, the absorbance was detected by an enzyme-linked immunosorbent assay plate reader (Epoch, BIO-TEK, USA, 450 nm), and the corresponding optical density (OD) value acquired was utilized for cell viability assessment.

### Cell migration assay

Transwell assay was used for evaluating the ability of cell migration. Transwell plate (Millipore) is divided into two chambers: the upper chamber and the lower chamber. In our study, the upper chamber was seeded with approximately 0.5 × 10^4^ HUVEC incubated with serum-free medium. Then, different treatments including LG, HG, HG + Exos (50 μg/mL), and HG + ATV-Exos (50 μg/mL) were added into the lower chamber. Being cultured in an incubator at 37 °C for 48 h, the culture medium was removed, and the cells were washed with PBS for three times. Subsequently, 4% paraformaldehyde was utilized for the fixation of cells for 15 min before stained with 0.1% (w/v) crystal violet for 7 min. Eventually, the migrated HUVECs were observed by an optical microscope (Olympus IX 70, Tokyo, Japan) and counted by ImageJ software.

### Tube formation assay

For ascertaining the capillary-like structure formation capacity of HUVEC under LG, HG, HG + Exos (50 μg/mL), and HG + ATV-Exos (50 μg/mL), tube formation assay was performed. Briefly, after the Matrigel™ was thawed in a 4 °C refrigerator overnight, the pre-cooling 24-well plate was loaded with the Matrigel™ at 200 μl/well and shaken evenly in ice, which avoided the untimely gelation of Matrigel™. Subsequently, a 24-well plate was placed in the culture incubator for 1 h for promoting the gelation of Matrigel™. Then, HUVEC at a density of 1.5 × 10^5^ per well was seeded onto the 24-well and incubated with culture medium of LG, HG, Exos, or ATV-Exos for 1 h. After 8 h, capillary-like structure formation was observed via an inverted light microscope and the formed capillary-like structure was counted by ImageJ.

### RNA extraction and qRT-PCR analysis

About 2 × 10^5^ HUVEC was seeded onto a 6-well plate and incubated with LG, HG, HG + Exos (50 μg/mL), and HG + ATV-Exos (50 μg/mL). TRIzol Reagent (Invitrogen) was utilized for the extraction of RNA. Then, total RNA was used for the generation of complementary DNA via RevertAid First-Strand cDNA Synthesis Kit (Takara). After that, qRT-PCR was conducted via ABI Prism 7300 Thermal Cycler (Applied Biosystems) with SYBR Green detection reagent (Takara). The results were normalized to the 18S gene. The relative expressions were calculated by formula 2^-(△△CT)^. All the primer sequences were listed as follows: PDGF (Forward, CTCGATCCGCTCCTTTGATGA; Reverse, CGTTGGTGCGGTCTATGAG); ANG1 (Forward, AGCGCCGAAGTCCAGAAAAC; Reverse, TACTCTCACGACAGTT-GCCAT); bFGF (Forward, AGAAGAGCGACCCTCACATCA; Reverse, CGGTTA-GCACACACTCCTTTG); EGF (Forward, TGTCCACGCAATGTGTCTGAA; Reverse, CATTATCGGGTGAGGAACAACC); 18S (Forward, GGACAGGATTGA-CAGATTGATAG; Reverse, CTCGTTCGTTTATCGGAATTAAC).

### Animal operation

All the animal procedures in this study got permitted to be conducted by the Animal Care and Experimental Committee of the Shanghai Jiao Tong University affiliated Sixth People’s Hospital. Thirty Sprague-Dawley rats (SD, 250 g ± 20 g, 8-week-old, male) were prepared for the operation. Streptozotocin (STZ, 65 mg/kg) was injected into all rats by intraperitoneal administration to generate diabetes models. We chose the eligible rats whose fasting blood glucose level was over 250 mg/dL for the following experiments. After being anesthetized by 0.6% pentobarbital sodium (10 ml/kg) via intraperitoneal injection, the SD rats received full-thickness skin excisions. Circular full-thickness wounds of 2 cm in diameter were generated on the back of the rats, and the wounds were treated with PBS, Exos, or ATV-Exos through multipoint injection (six points). After the operation, the defects were covered by a skin patch (3 M) and all the rats were sent back to the biosafety facility and gained free access to food and water. At day 0, 3, 7, and 14 postoperatively, the skin patch was removed, and a digital camera was applied to get the images of the recovery outcomes of the diabetic wounds. ImageJ (NIH Image) was used for the measurement and calculation of the wound dimension. The wound healing rates were calculated by formula [(C_0_-C_t_)/C_0_] × 100%. *C*_*0*_ is deemed the wound dimension at day 0, and *C*_*t*_ is the wound dimension at each time point.

### Microfil perfusion

Microfil perfusion was utilized for the evaluation of the neovascularization. The rib cages of the rats were opened after the anesthesia with 0.6% phenobarbital 14 days after the surgery. Having clamped the pulmonary artery and pulmonary vein, we used an indwelling needle for the penetration of the left ventricle. The 100-ml heparinized saline was injected for emptying the blood vessel through indwelling needle. Then, 15-ml microfil (Microfil MV-122; Flow Tech, Carver, MA) perfusate was injected into the left ventricle at a rate of 3 ml/min. After the perfusion, all the samples were immediately placed at 4 °C for the induction of microfil agent polymerization. After 24 h, the skin tissues around the wound sites were collected. Micro-CT (Skyscan 1176, Belgium) was applied to scan the samples at a resolution of 9 μm to observe neovascularization. Eventually, CTVox software was utilized for the analysis of the acquired images. The number of neovessels in the defect field was counted via ImageJ (NIH Image).

### Histology, immunohistochemistry, and immunofluorescence analysis

After the euthanasia by overdose pentobarbital sodium of rats, the wound tissues were collected and fixed in 4% paraformaldehyde at day 7 and 14 postoperatively. All the collected samples were dehydrated and embedded in paraffin. After the embedding, the paraffin block was sliced into 5-μm-thick sections. Hematoxylin and eosin (H&E) staining was utilized for observing the neuroepithelium length. Meanwhile, Masson’s trichrome staining was applied to observe the collagen synthesis.

For immunohistochemistry staining, the obtained paraffin sections were rehydrated. After that, the paraffin sections were incubated with CD31 primary antibody (1:200, Abcam) before treated with secondary antibody and ABC complex. Eventually, the samples were colored by DAB substrate. An optical microscope (Olympus IX 70, Tokyo, Japan) was applied to observe the stained sections.

For the assessment of the immunofluorescence. The paraffin sections were blocked by the 1.5% goat serum (Merck-Millipore) after being rehydrated. Alexa Fluor 488 as well as Cy3-conjugated secondary antibody and DPAI (Sigma-Aldrich) was used for the incubation for visualization after treated with the primary antibody CD31 (1:200, Abcam) and alpha-smooth actin (α-SMA) (1:50, Abcam). Then, an upright fluorescence microscope was applied to observe the stained sections in a dark room. For the counting of the new blood vessels, ImageJ (NIH Image) software was applied.

### Western blotting

For the verification of exosome and the mechanism of the pro-angiogenic ability by ATV-Exos, Western blotting was performed. Specifically, exosomes or cells were collected and lysed by RIPA. Next, 10% SDS-PAGE was utilized for the separation of proteins. Then, the PVDF membrane (Millipore) was applied to blot the proteins. Non-fat milk was used for the blocking of the membranes for 1 h. Subsequently, the PVDF membranes were incubated with primary antibodies at 4 °C overnight before treated with HRP-linked secondary antibodies on the next day for 1 h at room temperature. Finally, the protein bands were visualized via an ECL substrate kit (Merck Millipore, USA) and the expression level of proteins was quantified by ImageJ. All protein expression was normalized to HSP90 or GAPDH.

Antibodies against p-AKT (Ser473, 4060), eNOS (9586), p-eNOS (Ser1177, 9570), GAPDH (5174) and HSP90 (4877) were obtained from Cell Signaling Technology. Antibodies to Alix (12422-1-AP), TSG101 (14497-1-AP), CD81 (66866-1-Ig), PTEN (22034-1-AP), and AKT (10176-2-AP) were acquired from Proteintech.

### Statistics

All data were shown as mean ± standard error of the mean (SEM). One-way ANOVA was utilized for the evaluation of the significant difference. ^*^P value < 0.05 was considered significant. GraphPad Prism 8 software was appliedfor the statistical comparison of all the data.

## Results

### Characterization of BMSC-derived exosomes

**Fig. 1:**
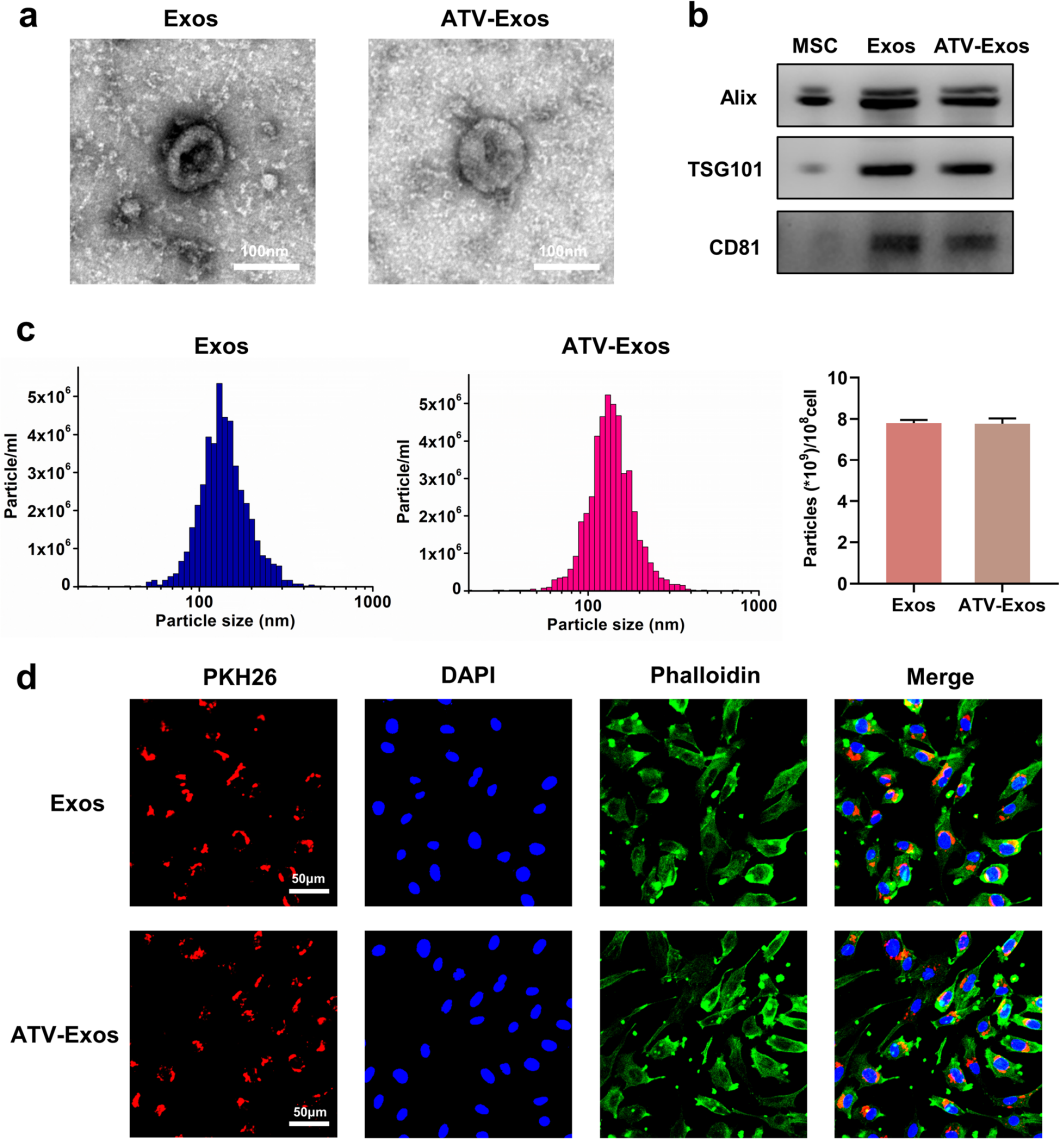
The characterization of BMSC-derived exosomes. **a** The morphology of Exos and ATV-Exos was examined by TEM. **b** The specific surface markers (Alix, TSG101, CD81) of Exos and ATV-Exos were assessed by Western blotting. **c** The diameter and particle concentration of Exos and ATV-Exos were detected via NTA. **d** The uptake verification of Exos and ATV-Exos by HUVEC via laser scanning confocal microscopy. Exosomes, cytoskeleton, and cell nucleus were stained red, green, and blue, respectively

We collected the supernatants of BMSC (no treatment) and ATV-treated BMSC to isolate Exos and ATV-Exos via ultracentrifugation, respectively. TEM, NTA, and Western blotting analysis were carried out to identify the harvested BMSC-derived exosomes. As shown in Fig. 1a, typical exosomal structures of homogeneous, spherical, and membrane-bound vesicles were observed in each group by TEM. The Western blotting analysis demonstrated that Alix, TSG101, and CD81 were detected in the isolated particles compared with the BMSC cells (Fig. 1b). Additionally, NTA suggested that the size distribution of Exos and ATV-Exos both displayed a single peak at approximately 80–120 nm (Fig. 1c). And these data indicated that the particle size, morphology, and protein concentrations were similar between Exos and ATV-Exos. In short, these results collectively confirmed the successful extraction of the exosomes. More importantly, the stimulation of ATV had no impact on the exosome secretion amount by BMSC.

Afterward, to clarify whether BMSC-exos could be endocytosed into HUVEC, exosomes labeled with PKH26 were co-cultured with HUVEC. In the picture of laser scanning confocal microscopy, PKH26-labeled exosomes (red) were localized in the perinuclear region of HUVEC (Fig. 1d), confirming the internalization of exosomes by endothelial cells.

### ATV-exos facilitated endothelial cell angiogenesis in vitro

**Fig. 2:**
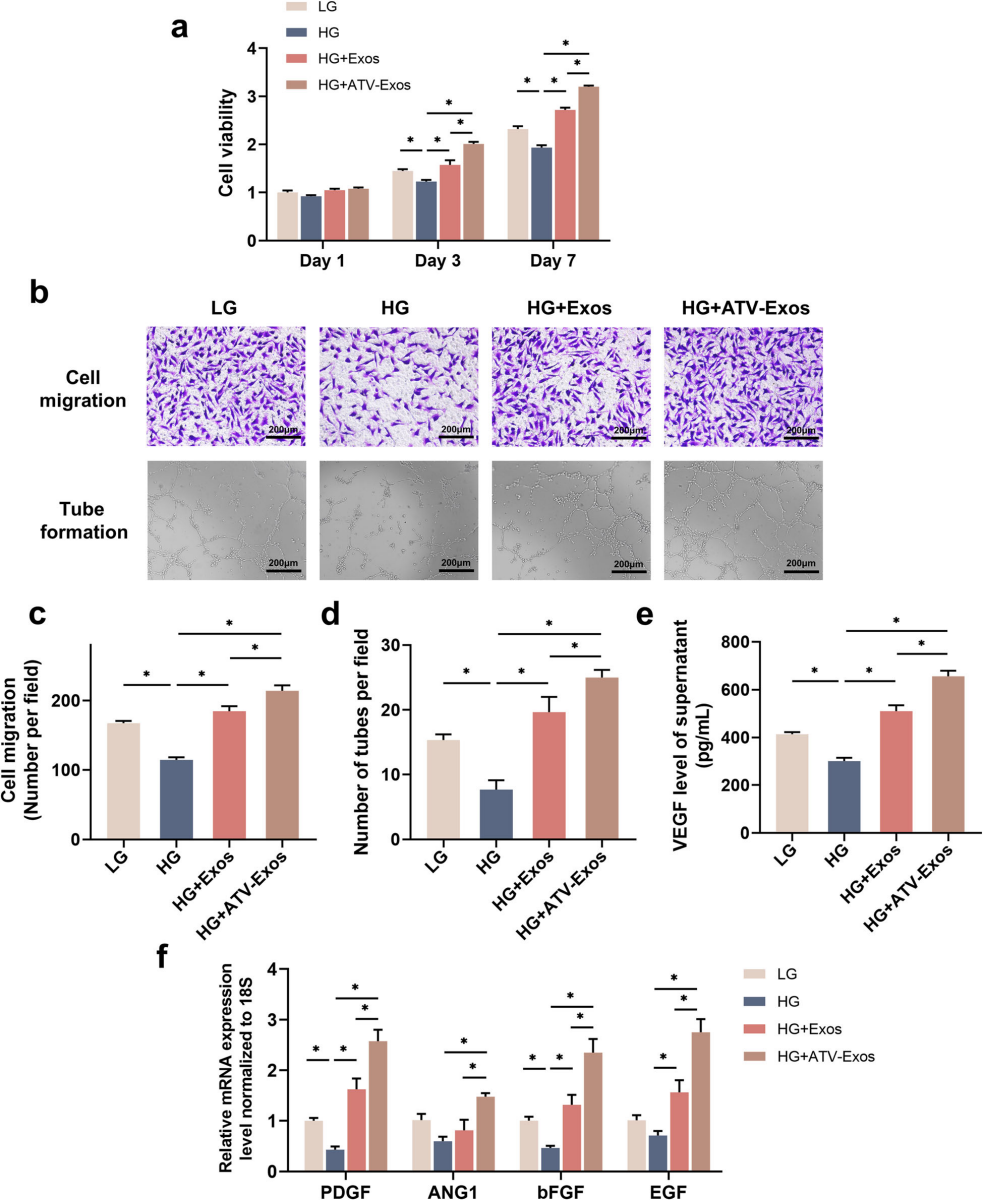
ATV-Exos facilitated endothelial cell angiogenesis in vitro. **a** The proliferation of HUVEC incubated with complete culture medium supplemented with LG, HG, HG + Exos, and HG + ATV-Exos for 1, 3, and 7 days. **b** The images of migration ability as well as tube formation of HUVEC. **c** Quantitative analysis of cell migration of four groups. **d** Quantitative analysis of tube formation of four groups. **e** The VEGF concentration of supernatant in different media supplemented with LG, HG, HG + Exos, and HG + ATV-Exos through Elisa. **f** The relative expression level of ANG1, PDGF, bFGF, and EGF of HUVEC examined by qRT-PCR. *P < 0.05

After HUVEC being treated with LG, HG, HG + Exos, and HG + ATV-Exos respectively, the CCK8 assay was performed for examining the effects of ATV-Exos on the cell proliferation of HUVEC. As demonstrated in Fig. 2a, Exos and ATV-Exos promoted the cell viability of endothelial cells impaired by HG at day 3 and 7 days while ATV-Exos exhibited a stronger pro-proliferative function than Exos. The role of ATV-Exos in the endothelial cell migration was evaluated by transwell assay, and the results suggested that there was an obvious increase in the motility of HUVEC in Exos and ATV-Exos groups compared with HG group. Moreover, ATV-Exos exhibited a greater promoting effect in cell migration (Fig. 2b, c). To investigate the pro-angiogenic capacity of ATV-Exos, we performed the tube formation tests. More tube structures in ATV-Exos-treated HUVEC were observed than that in the Exos and HG group, demonstrating enhanced tube formation ability of ATV-Exos (Fig. 2b, d). Meanwhile, we detected the effect of ATV-Exos on the proliferation and migration ability of fibroblasts and keratinocytes. Compared with the LG group, the other three groups decreased the cell viability significantly whereas no significant difference was observed between HG, HG + Exos, and HG + ATV-Exos group (Additional file 1: Figure S1).

Then, we utilized Elisa method to analyze the influence of ATV-Exos on VEGF secretion, which has been proved to induce angiogenesis and the formation of granulation tissue [22]. Exos and ATV-Exos increased the VEGF concentrations in the supernatants of HUVEC which were suppressed by HG treatment and ATV-Exos group showed more VEGF amount (Fig. 2e). Meanwhile, the expression levels of several other angiogenesis-related genes were assessed at mRNA levels and qRT-PCR results showed that ATV-Exos remarkably upregulated the level of PDGF, EGF, bFGF, and ANG1 in endothelial cells (Fig. 2f). Taken together, these results verified the potential of ATV-Exos in augmenting the biological functions of endothelial cells in vitro.

### ATV-Exos accelerated the wound healing of STZ-induced diabetic rats

**Fig. 3:**
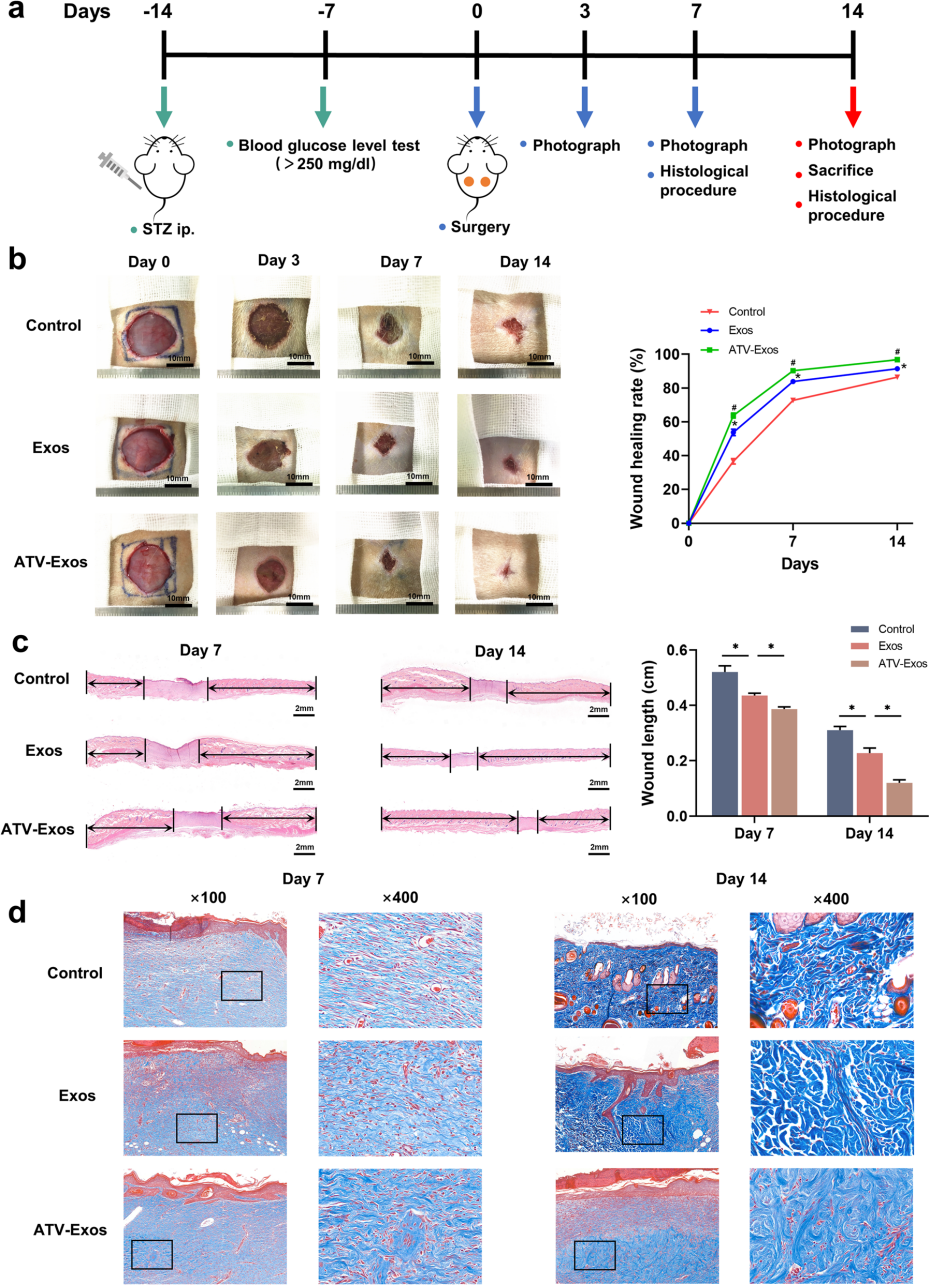
ATV-Exos accelerated the wound healing of STZ-induced diabetic rats. **a** Experimental design of the animal study. **b** Representative images of full-thickness defects and wound healing rates of the STZ-treated rats receiving a multipoint injection of PBS (control), Exo, and ATV-Exo at days 0, 3, 7, and 14 day postoperatively. *P < 0.05 versus control, ^#^*P* < 0.05 versus Exos. **c** H&E staining (black arrows indicate neuroepithelium) and quantification of wound length at day 7 and 14. Scale bars = 2 mm. **d** Masson’s trichrome staining at day 7 and 14 postoperatively at a magnification of × 100 and × 400. *P < 0.05

The procedures and timescale for developing and treating diabetic wound models were shown in Fig. 3a. Firstly, diabetic models were generated by injecting STZ into SD rats at a dose of 65 mg/kg and all rats were randomly divided into three groups, including control (PBS), Exos, and ATV-Exos groups. There was an apparent decline in the bodyweight with the blood glucose higher than 250 mg/dL 7 days after STZ injection while no significant difference was observed in the two indicators among the groups (Additional file 2: Figure S2).

Subsequently, we made full-thickness cutaneous skin defects of 2 cm in diameter on the back of the rats, being treated with multi-point subcutaneous injection of PBS (control), Exos, and ATV-Exos around the wound sites. From the images of skin defects, accelerated wound closure was observed in the Exos group compared with that in the control group while ATV-Exos exhibited more effective promotion at days 3, 7, and 14 postoperatively (Fig. 3b). No erythema, edema, or irritation were observed in the wound area with PBS, Exos, and ATV-Exos treatment during the whole period until the sacrifice. Besides, there was no elevation of renal damage indicators (creatine and BUN) and liver function markers (ALT and AST) in any group (Additional file 3: Figure S3), which confirmed the superior biosafety of the therapy.

In addition, compared with PBS and Exos, the diabetic wound of ATV-Exos group showed better reepithelization, as evidenced by more epithelial structures and longer neuroepithelium via optical micrographs of H&E staining (Fig. 3c). Masson staining represented that there were thicker wavy collagen fibers and more extensive collagen deposition arranged neatly in the ATV-Exos group, indicating its excellent ECM remodeling capacity (Fig. 3d). Collectively, these results revealed ATV pretreatment facilitated the BMSC-derived exosome-mediated diabetic wound closure in vivo with adequate security.

### ATV-Exos enhanced vascularization in response to a diabetic wound in vivo

**Fig. 4:**
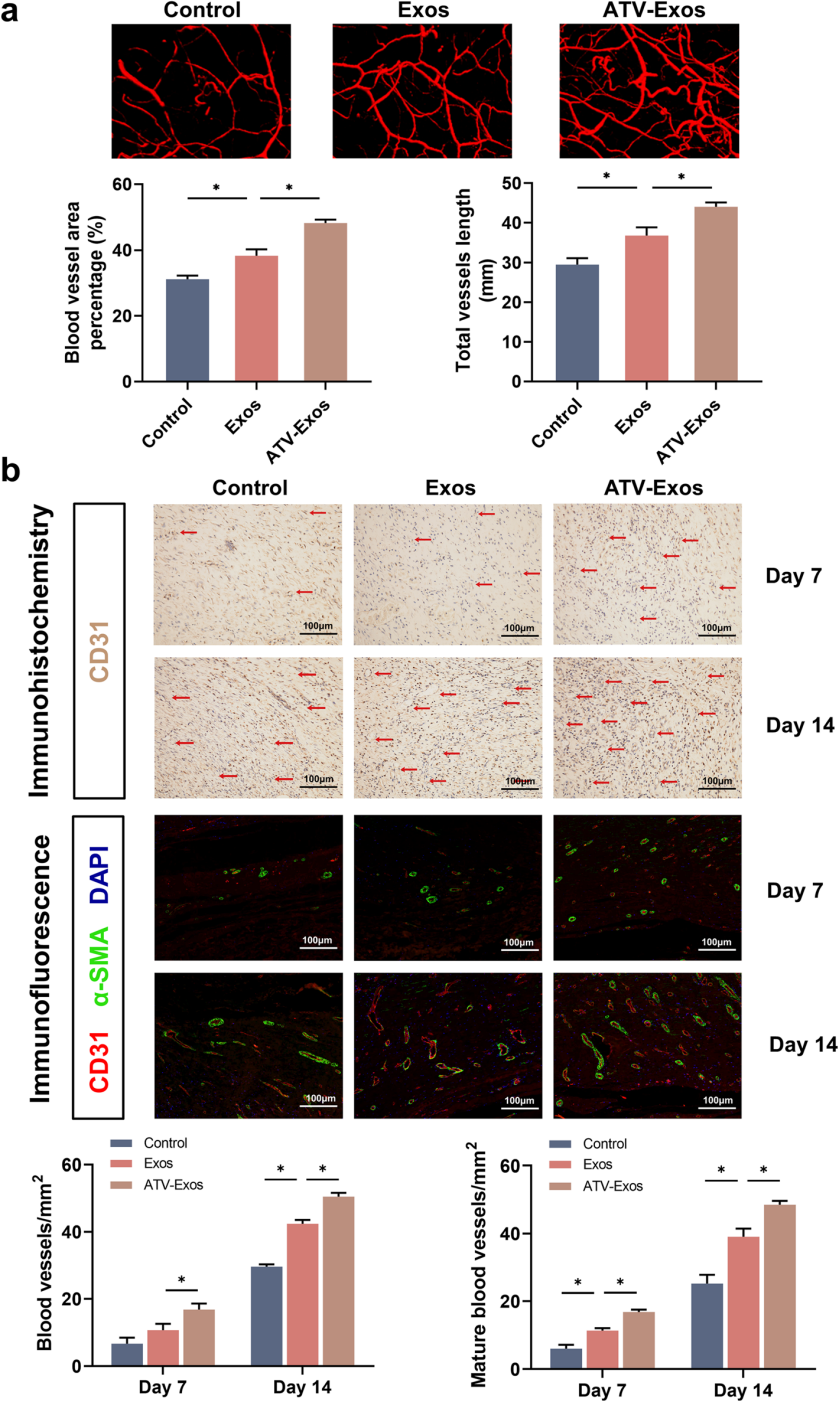
ATV-Exos ameliorated the aberrant vascularization of diabetic rats in vivo. **a** The representative images of microfil perfusion around the wound and quantitative analysis of vessel area and length in the control, Exo, and ATV-Exo groups. **b** Upper: Immunohistochemical analysis of newly developed vessels stained by CD31 at day 7 and 14 (red arrows represent vessels.). Scale bars = 100 μm. Middle: Immunofluorescence assessment of blood vessels stained by CD31/α-SMA at day 7 and 14. Lower: Quantitative analysis of blood vessel numbers per field by immunohistochemistry and immunofluorescence at days 7 and 14. *P < 0.05

To investigate the pro-angiogenic effects of ATV-Exos, microfil perfusion was carried out 14 days post-surgery and the three-dimension images suggested that wounds treated with Exos and ATV-Exos had much more blood vessel area and number compared with those in the control group. And, ATV-Exos induced more blood vessel formation than Exos (Fig. 4a).

CD31 and α-smooth muscle actin (α-SMA) are markers for detecting newly formed vessels and mature vessels, respectively [23]. Immunohistochemistry for CD31 as well as the immunofluorescence co-staining for CD31 and α-SMA together revealed that newly developed and mature blood vessels in the wound sites both increased after wounds being treated with ATV-Exos compared to that in control and Exos groups from day 7 to day 14. Quantitation of positive CD31 or α-SMA-stained cells also confirmed the conclusion (Fig. 4b). Hence, these data reflected the promotion of ATV-Exos on the number and maturity of blood vessels in diabetic skin defects.

### AKT/eNOS pathway was responsible for ATV-Exos-induced angiogenesis

**Fig. 5:**
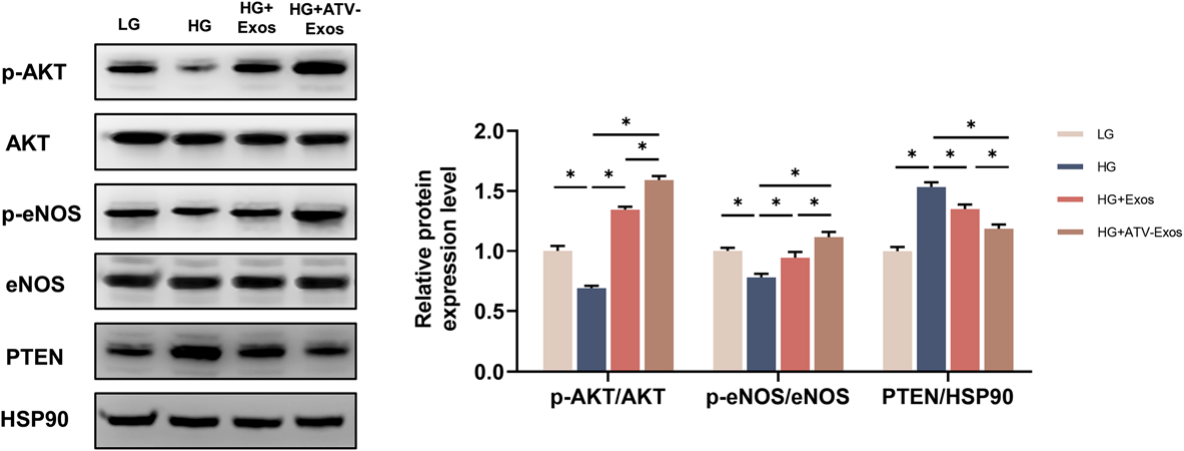
ATV-Exos activated angiogenesis-related AKT/eNOS pathway. HUVECs were treated with LG, HG, HG + Exos, and HG + ATV-Exos, and the protein levels of p-AKT, AKT, p-eNOS, eNOS and PTEN and HSP90 were explored by Western blotting with HSP90 being used as a control. *P < 0.05

The activation of the AKT/eNOS pathway has been proved to stimulate several crucial processes of angiogenesis, including endothelial cell survival, migration, and tube formation of endothelial cells [24]. Meanwhile, ATV could activate the AKT/eNOS pathway in several disease models like hindlimb ischemia and pulmonary arterial hypertension [25, 26]. What is more, previous studies have suggested that PTEN, a negative regulator of PI3K/AKT [27], was the potential target of ATV. Given that, we investigated whether the inhibition of PTEN and subsequent activation of the AKT/eNOS pathway were associated with ATV-Exos-mediated pro-angiogenesis. As shown in Fig. 5, Western blotting results revealed that the protein levels of phosphorylated AKT (p-AKT) and phosphorylated eNOS (p-eNOS) were downregulated by HG whereas Exos and ATV-Exos dramatically reversed this inhibition to activation states, which were more obvious in ATV-Exos group than that in Exos group. And, ATV-Exos apparently suppressed the protein level of PTEN.

**Fig. 6:**
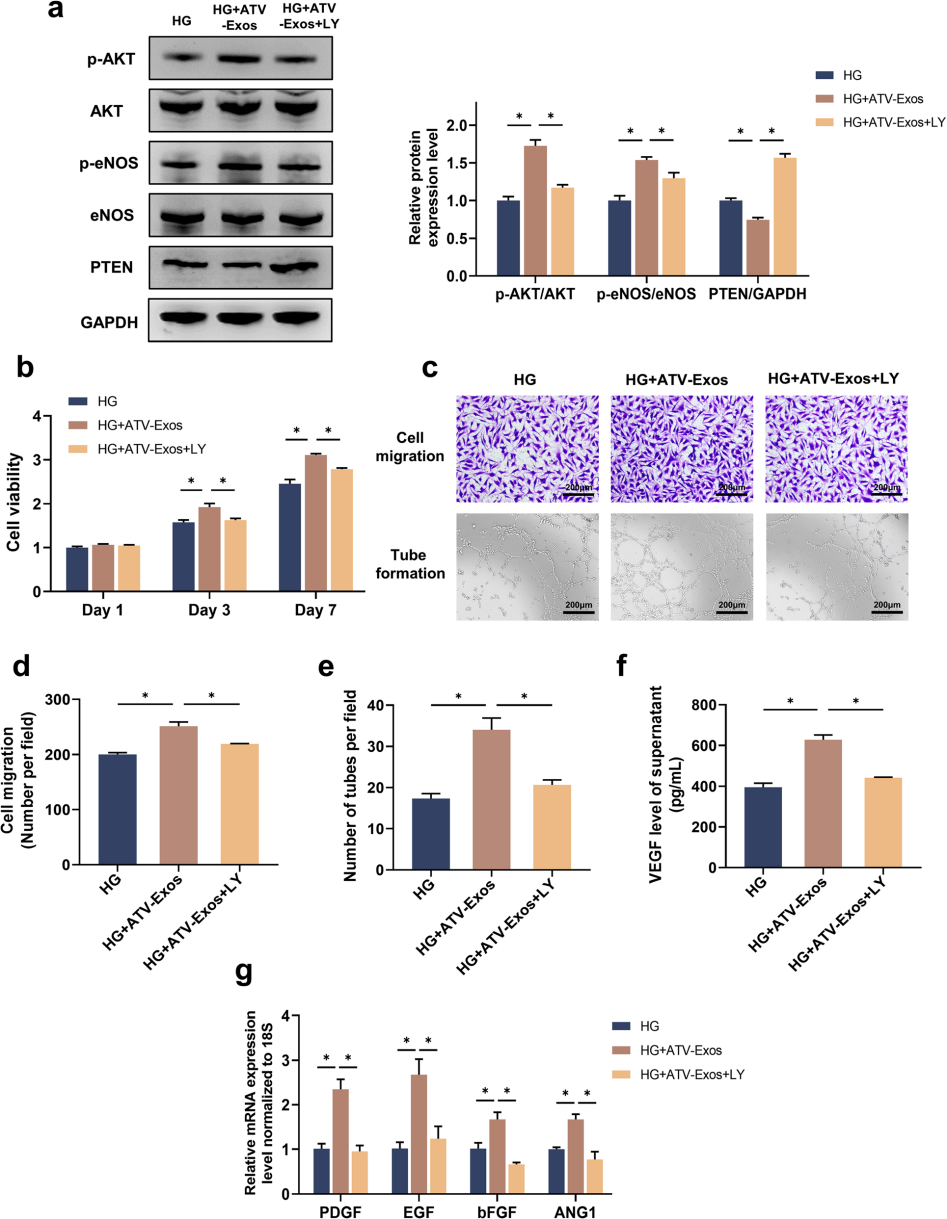
Proangiogenic capacity of ATV-Exos in HUVECs was attenuated when AKT/eNOS pathway was blocked. **a** The phosphorylation level of AKT and eNOS as well as the relative protein expression level of PTEN of HUVEC treated with HG medium supplemented with Exos, ATV-Exos, and ATV-Exos+LY 294002 by Western blotting. **b** Cell proliferation of HUVEC for 1, 3, and 7 days. **c** The images of cell migration and tube formation of HUVEC with different treatments. **d** Quantitative analysis of cell migration ability. **e** Quantitative analysis of tube formation ability. **f** The VEGF concentration of supernatant in HG medium supplemented with Exos, ATV-Exos, and ATV-Exos+LY 294002 through Elisa. **g** The relative expression level of ANG1, PDGF, bFGF, and EGF of HUVEC examined by qRT-PCR. LY: LY 294002. *P < 0.05

Next, to further elaborate on the role of AKT/eNOS signaling pathway in ATV-Exos promoted-angiogenesis, we used the inhibitor LY294002 to suppress the activation of AKT. Western blotting assay indicated that LY294002 attenuated the phosphorylation level of AKT and eNOS, and there was an increase in the level of PTEN as evidence by Fig. 6a. What stands out in Fig. 6b–f was that the enhanced cell proliferation, migration, tube formation, and VEGF level of ATV-Exos were partially blocked by LY294002, which also suppressed the increased mRNA levels of PDGF, EGF, bFGF, and ANG1 (Fig. 6g). Taken together, it was demonstrated that AKT/eNOS pathway played a vital role in the enhancement of endothelial cell functions by ATV-Exos.

### The inhibition of miR-221-3p suppressed ATV-Exos-mediated angiogenesis of endothelial cells

**Fig. 7:**
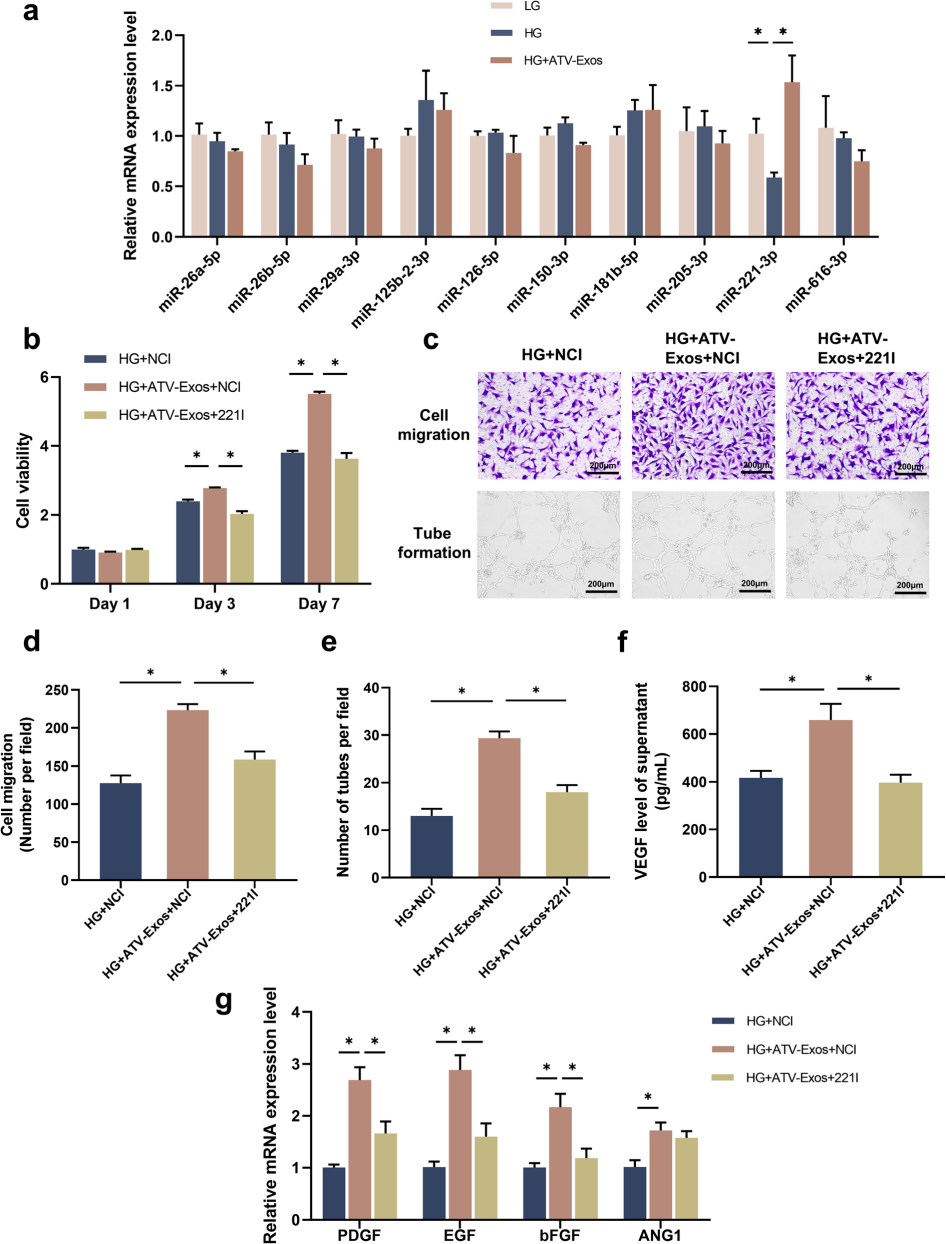
The inhibition of miR-221-3p suppressed ATV-Exos-mediated angiogenesis of HUVECs. **a** Ten candidate miRNAs were selected for qRT-PCR. **b** Cell proliferation of HUVEC treated by NCI, ATV-Exos+NCI, and ATV-Exos+221I for 1, 3, and 7 days. **c** The images of cell migration and tube formation of HUVEC treated with NCI, ATV-Exos+NCI, and ATV-Exos+221I. **d** Quantitative analysis of cell migration ability. **e** Quantitative analysis of tube formation ability. **f** The VEGF concentration of supernatant in HG medium supplemented with NCI, ATV-Exos+NCI, and ATV-Exos+221I through Elisa. **g** The relative expression levels of ANG1, PDGF, bFGF, and EGF of HUVEC treated with NCI, ATV-Exos+NCI, and ATV-Exos+221I examined by qRT-PCR. *P < 0.05

Many researches have confirmed the crucial role of miRNAs in the biological functions of endothelial cells during the wound repair and tissue regeneration process [28, 29]. To explore the underlying mechanism of PTEN/AKT/eNOS pathway in ATV-Exos-mediated pro-angiogenic capacity, we specifically selected ten candidate miRNAs which have been proved to regulate endothelial cell function and could also activate PTEN/AKT pathway. The qRT-PCR results revealed that ATV-Exos remarkably upregulated the level of miR-221-3p which was inhibited by HG, whereas no significant change was observed in the expressions of miR-26a-5p, miR-26b-5p, miR-29a-3p, miR-125b-2-3p, miR-126-5p, miR-150-3p, miR-181b-5p, miR-205-3p, and miR-616-3p among different groups (Fig. 7a). Subsequently, to further explore whether miR-221-3p mediated the enhanced angiogenesis of ATV-Exos, we performed miR-221-3p inhibition by transfection with miR-221-3p inhibitor (221I) and negative control inhibitor (NCI) in HUVEC.

CCK8 and transwell assays demonstrated that the cell viability and migration rate of HUVEC treated with 221I were remarkably inhibited compared with the NCI (Fig. 7b–d). Meanwhile, the tube formation assay showed that the number of tube-like structures decreased sharply in the HG-ATV-Exo + 221I group (Fig. 7c, e). The VEGF secretion was also markedly attenuated by 221I (Fig. 7f). Meanwhile, the mRNA levels of PDGF, EGF, and bFGF were downregulated by 221I, but it has no significant influence on the expression of ANG1 (Fig. 7g). Above all, this study indicated that ATV-Exos upregulated the level of miR-221-3p in HUVEC and the inhibition of miR-221-3p gave rise to impaired proliferation, migration, tube formation, and VEGF secretion in endothelial cells.

## Discussion

Delayed diabetic wound healing has already become an intractable medical problem whereas the current strategies are function-limited. In this study, we explored the pro-angiogenic property of exosomes extracted from BMSCs pretreated with ATV to accelerate the impaired diabetic wound healing in vivo and in vitro. We also made an inquiry into the possible underlying mechanism. Our results illustrated that ATV-Exos activated AKT/eNOS signaling pathway to augment the angiogenesis of endothelial cells via upregulating miR-211-3p, thereby resulting in accelerated wound regeneration of the diabetic rats. These findings suggested that ATV preconditioning is a promising candidate to enhance the therapeutic efficacy of MSC-Exos-based strategy for diabetic skin defects.

By transporting nutrients, oxygen, and growth factors to the injury sites, the formation of new blood vessels is essential for skin tissue regeneration during the wound healing [10]. However, the dysfunction of endothelial cells in diabetes contributes to the decline in angiogenesis, characterized by decreased vascularity and capillary density. In turn, the wound repair is delayed as a result of insufficient angiogenesis [30]. Though MSCs have been reported of the beneficial effects on the cutaneous regeneration during wound repair, there are still many drawbacks hindering their clinical practice, such as immunogenicity and tumorigenic potential. Compared with MSCs, MSC-Exos have more intense biological functions and are more stable resulting from their plasma membrane which are less easily destroyed. More importantly, MSC-Exos have pretty lower risks of tumorigenesis, immune rejection, and pulmonary embolism [31, 32]. Over the last few years, researchers spared no effort to develop modified MSC-Exos to get more superior effects on the wound healing. Yang et al. [33] demonstrated that exosomes derived from blue light stimulated-human umbilical cord MSC improved wound healing via upregulating MEF2C signaling. Another literature by Ding et al. [34] indicated that deferoxamine stimulated the cutaneous wound repair of MSC-Exos by increasing angiogenesis in STZ-induced diabetic rat model. Although these small molecule compounds appear to have beneficial effects on wound regeneration, they are still less feasible in clinical application in a short time owing to their unverified safety. Instead, ATV has been one of the most widely prescribed drugs in the world to decreases blood cholesterol clinically [35] and this study also revealed the drug has excellent biosafety when being administered in wound healing. Meanwhile, ATV-Exos was capable of accelerating the skin tissue repair of diabetic cutaneous wound dramatically according to the results. Therefore, our findings confirmed that ATV-Exos has immediate translational potential for treating the diabetes patients suffered from hard-to-heal ulcers.

ATV has been proved to promote the tissue repair in acute lesion rat model independently of its lipid-lowering action [20]. Similarly, a recent study by Morsy et al. [36] suggested that nanoemulgel loaded with ATV accelerated the tissue repair of skin defects. Nevertheless, ATV may give rise to muscle pain and weakness whereas these side effects could be effectively circumvented by ATV-Exos treatment.And ATV-Exos has been reported by researchers to play therapeutic roles in different disease models. For example, in acute myocardial infarction rats, the effects of MSC-derived exosome were enhanced by ATV preconditioning in protecting cardiomyocytes, increasing angiogenesis, and improving cardiac function [21]. ATV-Exos has also been reported to moderate autoimmune myasthenia gravis via upregulating IDO/Treg pathway [37].

Our study firstly demonstrated that ATV pretreatment enhanced the therapeutic efficacy of MSC-derived exosomes in promoting angiogenesis. The in vitro results revealed that ATV-Exos significantly improved the biological function of endothelial cells including their proliferation, migration, tube formation, and VEGF secretion which were damaged by HG. Furthermore, the relative expression levels of angiogenesis-associated genes (PDGF, bFGF, EGF, and ANG1) were upregulated in HUVEC cultured with ATV-Exos. FGF, PDGF, and bFGF have been reported of facilitating vascularization and granulation tissue formation, leading to accelerated re-epithelialization and wound regeneration [38]. Likewise, ANG1 could promote endothelial cell proliferation and migration [39]. In vivo, microfil, immunohistochemistry, and immunofluorescence results collectively indicated that ATV-Exos treatment of the skin defects did not only increase the number of newly developed blood vessels but also improve their maturity. 

In this study, we also investigated the underlying molecular mechanisms of ATV-Exos stimulated angiogenesis in vitro. Previous researches have verified that AKT exerted a critical role in angiogenesis by increasing endothelial repair and regeneration [40]. In addition, AKT was able to activate endothelial eNOS to increase NO production, protecting endothelial cell apoptosis induced by HG [24]. Meanwhile, PTEN could reverse the phosphorylation of the cytoplasmic PI3K/AKT pathway to inhibit AKT signaling and suppress AKT-mediated VEGF gene transcription as well as the migration and invasion of endothelial cells [41]. Our stusy showed that ATV-Exos groups activated AKT/eNOS pathway significantly. For further clarifying the possibly important effect of the AKT/eNOS signaling pathway on the effects of ATV-Exos, the results demonstrated that ATV-Exos no longer exhibited a distinct advantage over Exos in angiogenesis-promotion with LY294002 blocking the pathway according to the in vitro angiogenesis analysis. Hence, we can draw the conclusion that the better pro-angiogenic ability of ATV-Exos than Exos was mediated by the PTEN/AKT/eNOS pathway.

**Fig. 8:**
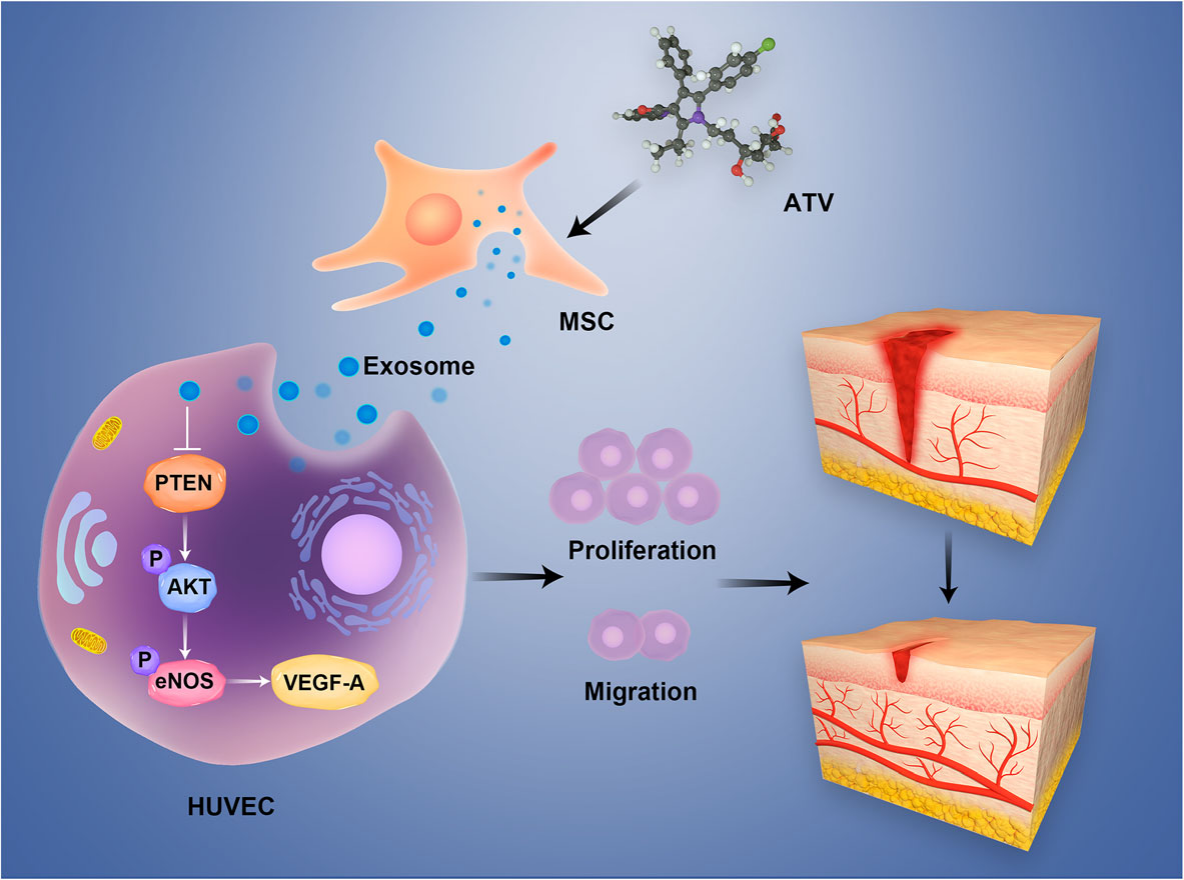
Schematic representation depicts that ATV-Exos could promote wound healing by activating AKT/eNOS pathway.

In this research, there was little significant difference in morphology, structure, concentration, and surface marker expression between ATV-Exos and Exos, so we can come to the conclusion that the outstanding pro-angiogenic capacity of ATV-Exos might result from the change in the amounts or activities of their cargoes, some bioactive molecule such as miRNAs, which was independent of the number of released exosomes. Previous study found that exosomes overexpressing miR-126 have been proved effective in diabetic wound repair [42]. Lithium could stimulate the secretion of exosome incorporated with miR-130a and thus could promote the angiogenesis [43]. Our results demonstrated that the changes of expression levels of miR-221-3p were responsible for the pro-angiogenic effect of ATV-Exos. Also, some researches have reported that the downregulation of miR-221-3p resulted in the impaired heart repair due to the attenuated angiogenesis ability [44]. The phosphorylation of AKT plays an important role in angiogenesis and PTEN, the direct target of miR-221, is the inhibitor of AKT while the downregulation of miR-221 could significantly inhibit the function of HUVEC, further impairing angiogenesis [45–47]. In this study, ATV-Exos markedly upregulated the level of miR-221-3p which was suppressed by HG and the treatment of miR-221-3p inhibitor significantly suppressed the promotion of endothelial cell proliferation, migration, and tube formation induced by ATV-Exos. Therefore, we believed that ATV-Exos could promote the angiogenesis ability of HUVEC by the upregulation of miR-221-3p via AKT/eNOS pathway (Fig. 8).

## Conclusion

In conclusion, this study firstly proved that ATV pretreatment enhanced the function of exosomes originated from MSC in promoting angiogenesis via upregulating the AKT/eNOS signaling pathway, leading to accelerated diabetic wound repair and regeneration. Our results offer a new perspective for the treatment of a diabetic wound as a cell-free therapy.

## Supplementary information

**Additional file 1: Supplemental Figure 1.** ATV-Exos have no impact on the proliferation and migration ability on fibroblasts and keratinocytes. **a** The proliferation of HFF-1 and HaCaT incubated with complete culture medium treated with LG, HG, HG+Exos, and HG+ATV-Exos for 1, 3, and 7 days. **b** The images of the migration ability of HFF-1 and HaCaT treated with LG, HG, HG+Exos, and HG+ATV-Exos. *P < 0.05.

**Additional file 2: Supplemental Figure 2.** The body weight and fasting blood glucose of diabetic rats. **a** The bodyweight of diabetic rats treated with PBS, Exos, and ATV-Exos before and after STZ injection, respectively. **b** The fasting blood glucose of diabetic rats in each group.

**Additional file 3: Supplemental Figure 3.** The hepatic and renal (creatine and BUN) function of diabetic rats. After 14 days post-surgery, serum ALT, AST (hepatic function), creatine, and BUN (renal function) were measured in control, Exos, and ATV-Exos groups.

## Data Availability

The datasets used and/or analyzed during the current study are available from the corresponding author on reasonable request.

## Abbreviations

α-SMA: α-Smooth muscle actin
ATV: Atorvastatin
ATV-Exos: Exosomes from atorvastatin-pretreated BMSC
Exos: Exosomes from non-pretreated BMSC
BMSC: Human bone marrow mesenchyme stem cell
HUVEC: Human umbilical vein endothelial cells
MSC: Mesenchyme stem cell
STZ: Streptozotocin
NTA: Nanoparticle tracking analysis
OD: Optical density
TEM: Transmission electron microscope
VEGF: Vascular endothelial growth factor
