# Supplementary material for: Exosomes derived from atorvastatin-pretreated MSC accelerate diabetic wound repair by enhancing angiogenesis via AKT/eNOS pathway
Source: Stem Cell Res Ther. 2020 Aug 12;11:350. doi: 10.1186/s13287-020-01824-2 (PMC7425015; doi:10.1186/s13287-020-01824-2)
Supplement: Supplementary file 1 — Additional file 1: Supplemental Figure 1. ATV-Exos have no impact on the proliferation and migration ability on fibroblasts and keratinocytes. a The proliferation of HFF-1 and HaCaT incubated with complete culture medium treated with LG, HG, HG+Exos, and HG+ATV-Exos for 1, 3, and 7 days. b The images of the migration ability of HFF-1 and HaCaT treated with LG, HG, HG+Exos, and HG+ATV-Exos. *P < 0.05. [file 13287_2020_1824_MOESM1_ESM.docx]

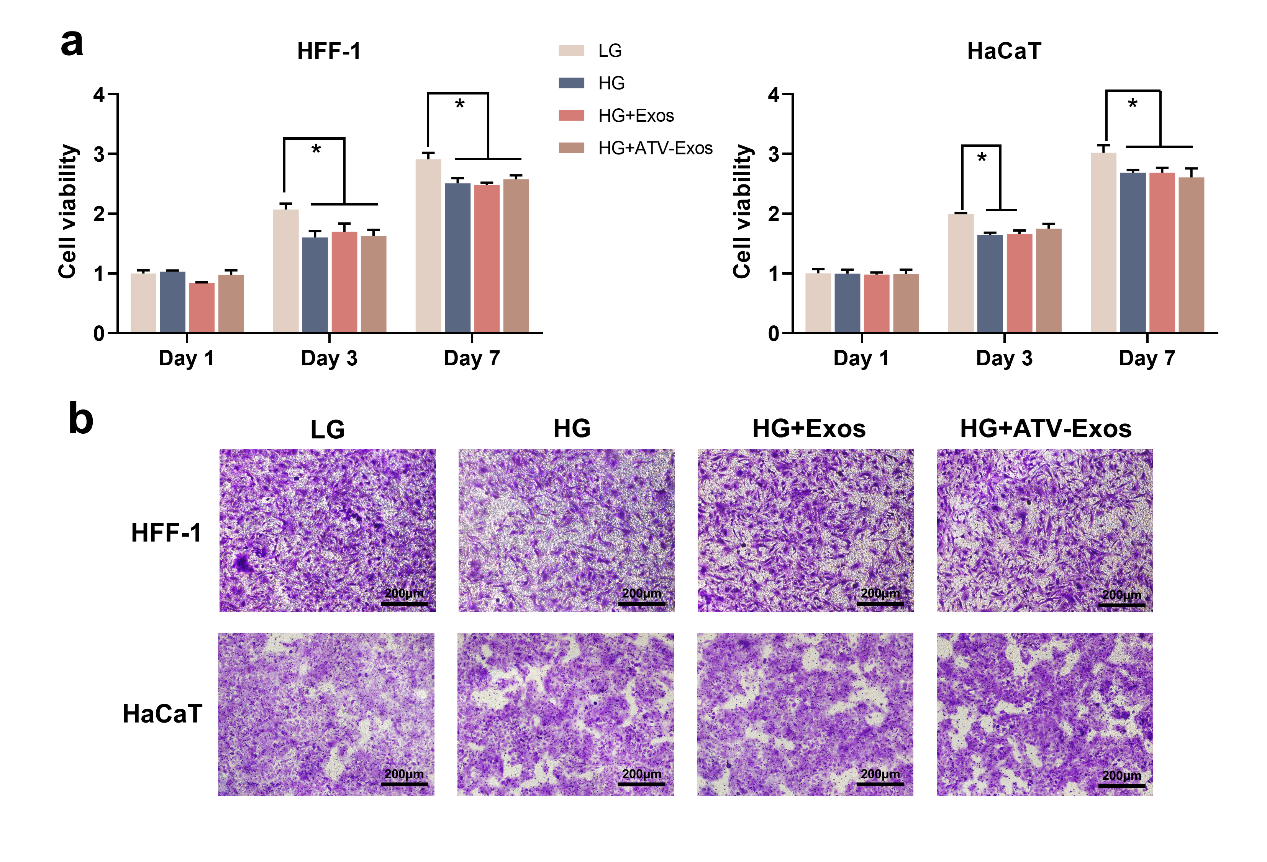
**Supplemental Figure 1. ATV-Exos have no impact on the proliferation and migration ability on fibroblasts and keratinocytes. a** The proliferation of HFF-1 and HaCaT incubated with complete culture medium treated with LG, HG, HG+Exos and HG+ATV-Exos for 1, 3 and 7 days. **b** The images of migration ability of HFF-1 and HaCaT treated with LG, HG, HG+Exos and HG+ATV-Exos. **P* < 0.05.
