# Supplementary material for: Exosomes derived from atorvastatin-pretreated MSC accelerate diabetic wound repair by enhancing angiogenesis via AKT/eNOS pathway
Source: Stem Cell Res Ther. 2020 Aug 12;11:350. doi: 10.1186/s13287-020-01824-2 (PMC7425015; doi:10.1186/s13287-020-01824-2)
Supplement: Supplementary file 2 — Additional file 2: Supplemental Figure 2. The body weight and fasting blood glucose of diabetic rats. a The bodyweight of diabetic rats treated with PBS, Exos, and ATV-Exos before and after STZ injection, respectively. b The fasting blood glucose of diabetic rats in each group. [file 13287_2020_1824_MOESM2_ESM.docx]

**
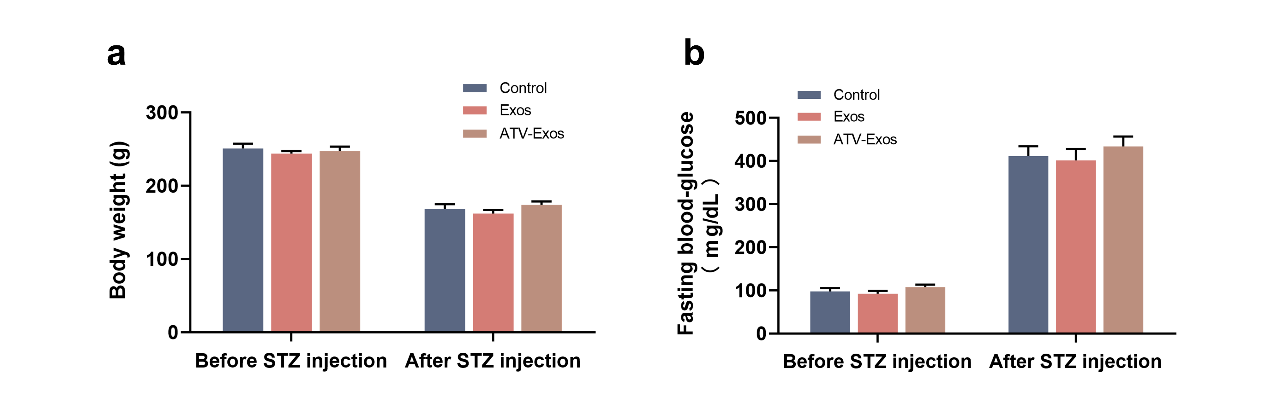
**

**Supplemental Figure 2. The body weight and fasting blood glucose of diabetic rats. a** The body wight of diabetic rats treated with PBS, Exos and ATV-Exos before and after STZ injection, respectively. **b** The fasting blood glucose of diabetic rats in each groups.
