# Supplementary material for: Exosomes derived from atorvastatin-pretreated MSC accelerate diabetic wound repair by enhancing angiogenesis via AKT/eNOS pathway
Source: Stem Cell Res Ther. 2020 Aug 12;11:350. doi: 10.1186/s13287-020-01824-2 (PMC7425015; doi:10.1186/s13287-020-01824-2)
Supplement: Supplementary file 3 — Additional file 3: Supplemental Figure 3. The hepatic and renal (creatine and BUN) function of diabetic rats. After 14 days post-surgery, serum ALT, AST (hepatic function), creatine, and BUN (renal function) were measured in control, Exos, and ATV-Exos groups. [file 13287_2020_1824_MOESM3_ESM.docx]

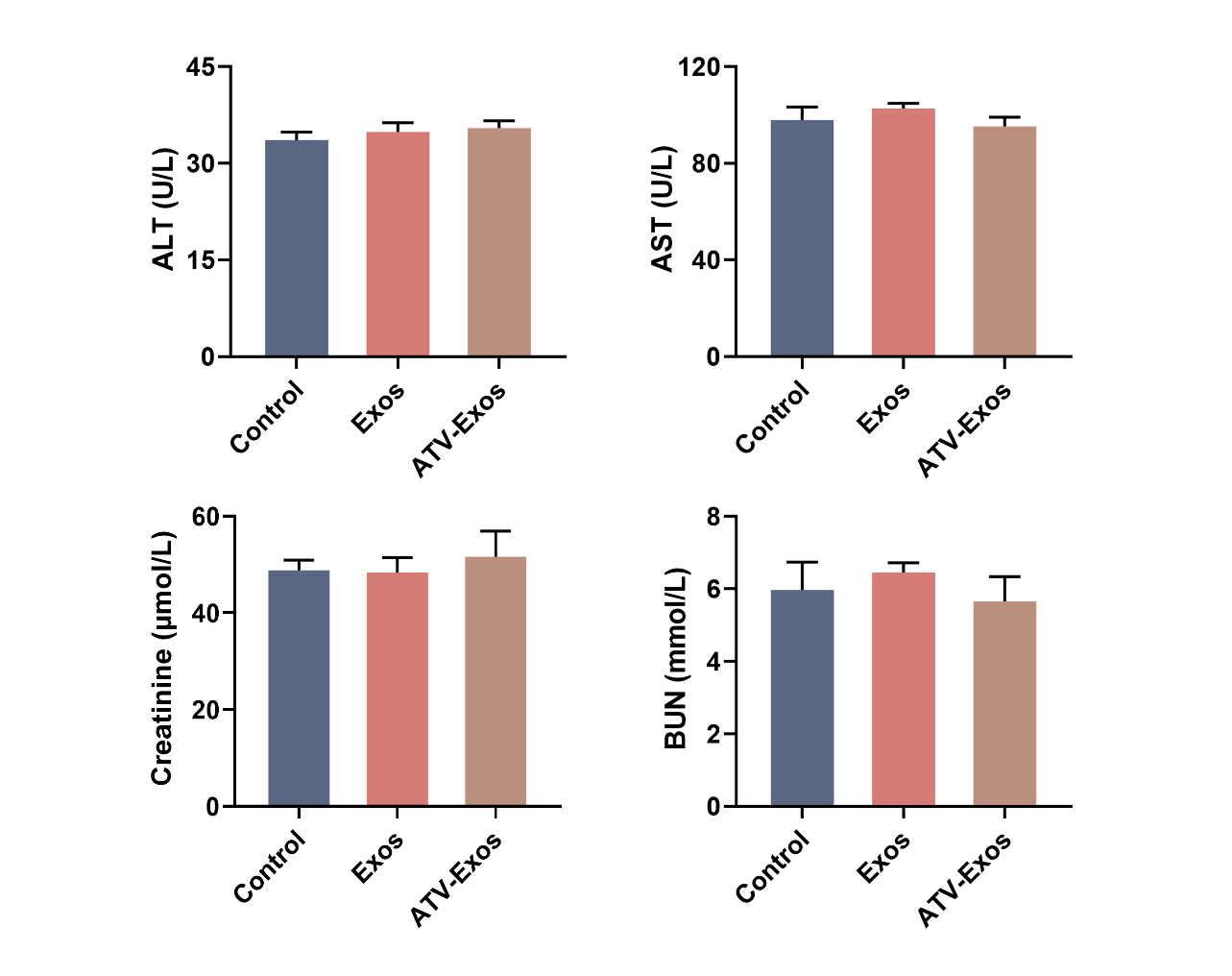


**Supplemental Figure 3. The hepatic and renal (creatine and BUN) function of diabetic rats.** After 14 days post-surgery, serum ALT, AST (hepatic function), creatine and BUN (renal function) were measured in control, Exos and ATV-Exos groups.
